# Overexpression of miRNA-3613-3p Enhances the Sensitivity of Triple Negative Breast Cancer to CDK4/6 Inhibitor Palbociclib

**DOI:** 10.3389/fonc.2020.590813

**Published:** 2020-11-27

**Authors:** Yuanhang Yu, Han Liao, Rong Xie, Yue Zhang, Renjing Zheng, Jianying Chen, Bo Zhang

**Affiliations:** ^1^Department of Breast and Thyroid Surgery, Union Hospital, Tongji Medical College, Huazhong University of Science and Technology, Wuhan, China; ^2^Department of Gastrointestinal Surgery, Union Hospital, Tongji Medical College, Huazhong University of Science and Technology, Wuhan, China

**Keywords:** CDK4/6 inhibitor, triple negative breast cancer, microRNA, cell cycle, EZH2, senescence

## Abstract

Triple negative breast cancer (TNBC) is characterized by lack of expression of the estrogen and progesterone receptors and HER2, which are common therapeutic targets. CDK4/6 inhibitor Palbociclib has been approved as an anti-cancer agent for breast cancer. However, identifying biomarkers that predict the response to Palbociclib has always been a challenge for molecular targeted therapy. In this study, we identify microRNA as a hallmark in TNBC patients and explore if miR-3613-3p might serve as a tumor suppressor biomarker for triple negative breast cancer patients and if overexpression of miR-3613-3p could enhance the sensitivity of TNBC cells to Palbociclib. We show that the expression of miR3613-3p was down-regulated in TNBC tumors and cells, and the overexpression of miR-3613-3p in patients’ tumor tissues was clinically and pathologically correlated with favorable prognosis, such as smaller tumor size and the lower Ki-67. *In vitro*, overexpression of miR-3613-3p inhibited cell proliferation, induced G1 cell-cycle arrest, and enhanced the sensitivity of TNBC cells to Palbociclib treatment. *In vivo* study revealed that overexpression of miR-3613-3p inhibited TNBC tumorigenesis and exerted a significant inhibitory effect of Palbociclib on MDA-MB-231 cells. Mechanically, SMAD2 and EZH2 were found to be two direct targets of miR-3613-3p and mediate the proliferation of TNBC cells and the sensitivity of the cells to Palbociclib through inducing cellular senescence. Our findings suggested that miR-3613-3p acts as a cancer-suppressor miRNA in TNBC. Moreover, our study showed that miR-3613-3p might be used as a predictive biomarker for the response of TNBC to Palbociclib.

## Introduction

Breast cancer remains the most common cancer and the second leading cause of cancer death in women, after lung cancer (1) and is the primary cause of cancer deaths in women (2). Breast cancer represents a heterogeneous disease with distinct biological subtypes (3). The triple negative breast cancer (TNBC) neither expresses the two hormone receptors nor expresses HER2 and its presence is associated with the worst prognostic outcome (4). TNBC is characterized by an increased likelihood of recurrence, distant metastasis, and mortality within 5 years of diagnosis, with the highest incidence being in the first 3 years (5). Unlike other breast tumor subtypes, it doesn’t express readily detectable receptors; anti-tumor agents specifically targeting TNBC have not yet been available (6). Therefore, many researchers are looking for specific targeted drugs to treat TNBC (7), but no significant progress has been made so far.

Palbociclib (PD 0332991) is a potent and highly selective inhibitor of CDK4 and CDK6 kinases (8). As an orally given agent for the first-line treatment of advanced post-menopausal ER+, HER2-negative breast cancer, used in combination with letrozole, was approved by the US Food and Drug Administration (FDA) in 2015 (9). A general consensus has been reached about the early use of a CDK 4/6 inhibitor in younger patients and in those with aggressive, PR-negative and/or symptomatic malignancies (10). Patients with triple-negative disease also benefited from this therapy (11). The activity of Palbociclib as a single agent in unselected patients with metastatic breast cancer was 19%, and all patients who clinically responded to the therapy had estrogen receptor-positive disease, though one of our patients with triple-negative disease also benefited from this therapy (12).

MicroRNAs are small non-coding endogenous RNA molecules, which are integral elements in the post-transcriptional control of gene expression. Mechanistically, miRNA can bind to the 3′UTR of target mRNAs, resulting in translational repression or target mRNA cleavage (13). Multiple studies have demonstrated that miRNAs are closely associated with the initiation, promotion, and progression of breast cancer, and miRNAs are implicated in the pathological processes of breast cancer, such as cell proliferation, metastasis, migration, and DNA methylation and so on (14). Both microRNA profile and gene studies have recently been carried out to dissect the different molecular entities in TNBC, which is a very heterogeneous disease where different subgroups can be recognized. Moreover, several microRNAs crucially involved in TNBC biology have been identified, providing the experimental basis for the potential therapeutic application and use of these small non-coding RNA molecules as biomarkers (15).

SMAD Family Member 2 (SMAD2) proteins act as the key components downstream of TGF-*β* signaling and regulate the transcriptional response for TGF-*β* functions (16). SMAD2 is overexpressed in breast cancer tissues and plays an important part in the promotion of tumor progression. It has been shown that inhibitors that decrease Smad2 and Smad3 levels by interrupting TGF-*β* signaling might be used for the treatment of human cancers, such as breast cancer and glioblastoma (17, 18). Recently, miRNAs have been identified and shown to inhibit tumor growth and migration by regulating SMAD2. For example, miR-27a inhibits colon cancer cell proliferation, promotes apoptosis, and attenuates cell migration (19). The Enhancer of Zeste Homolog 2 (EZH2) is a member of the Polycomb group (PcG) protein family, which, as a methyltransferase, plays a critical role in the regulation of cell proliferation, migration, stem cell fate, and tumorigenesis. Recent studies have demonstrated that EZH2 contributes to carcinogenesis by acting as an oncogene. Strong EZH2 expression was associated with increased tumor cell proliferation in many cancer types, including astrocytoma, breast cancer, prostate cancer, bladder cancer, hepatocellular carcinoma, colon cancer, lung cancer, and pancreatic cancer (20). More and more studies have reported that EZH2 can be regulated by non-coding RNAs, including miRNAs (21).

In this study, we compared the miRNA expression profiles in breast cancer tumors and adjacent normal tissues by miRNA array analysis. We found that miR-3613-3p was significantly decreased in both tumor tissues of TNBC patients and TNBC cell lines. In addition, overexpression of miR-3613-3p markedly inhibited the proliferation and migration in TNBC cells, partially through targeting SMAD2 and EZH2 *in vitro* and *in vivo*. More importantly, we demonstrated that overexpression of miR-3613-3p enhanced the sensitivity of Palbociclib treatment in TNBC both *in vitro* and *in vivo*. Our findings suggested that miR-3613-3p serves as a cancer-suppressor miRNA and functions as an anti-cancer gene. Overexpression of miR-3613-3p may be a novel strategy for inhibiting breast cancer proliferation and may be a predictive biomarker for the therapeutic use of Palbociclib in TNBC.

## Material and Methods

### Clinical Samples

Forty-four breast cancer samples and their adjacent normal breast tissues were collected from breast cancer patients who had undergone surgery at the Department of Breast and Thyroid Surgery, Union Hospital, Tongji Medical College, Huazhong University of Science and Technology, Wuhan, China. The study was approved by the institutional human Ethics Committee of Union Hospital, Tongji Medical College, Huazhong University of Science and Technology. Samples were immediately snap-frozen in liquid nitrogen and stored at −80°C for later use.

### Cell Lines

Human breast cancer cell lines, MDA-MB-231, SKBR3, T47D, BT474, and MCF7, were obtained from the American Type Culture Collection. Human breast cancer cell line MDA-MB-468 and the non-tumor cell line MCF10A were from Guangzhou Cellcook Biotech. These cell lines were cultured in recommended media. Cell line authentication was performed by STR analysis.

### Transfection of microRNA Mimics and Inhibitors

MiRNA mimics and miRNA inhibitors were designed and synthesized by Guangzhou RiboBio (RiboBio, China). MDA-MB-231 and MDA-MB-468 cells were seeded at 20 × 10^4^ cells per well into 6-well plates and transfected with 10 nM miRNA-3613-3p mimics, or the appropriate miRNA mimic control using Lipofectamine3000 transfection reagent (Thermo Fisher). MCF10A cells were seeded at 10 × 10^4^ cells per well into 6-well plates and transfected with microRNA-3613-3p inhibitors at a final concentration of 10 nM or the appropriate miRNA inhibitor control using Lipofectamine3000 transfection reagent (Thermo Fisher). Total RNA and protein were collected for assay 48–72 h after transfection.

### RNA Extraction

Total RNA was extracted from tissues or cell lines by using TRIzol reagent (Invitrogen) by the following instructions. MicroRNAs were efficiently isolated using the RNAiso for Small RNA Kit (9753A, Takara). The concentration, purity, and amount of total RNA were determined on the Nanodrop 1000 spectrophotometer (Nanodrop Technologies Inc. USA).

### Stem-Loop Reverse Transcription and Real-Time Polymerase Chain Reaction

A total of 1 μg of total RNA was reverse-transcribed in a stem-loop reverse transcription (RT) reaction by using RT primers and the PrimeScript™ RT reagent kit according to the user’s manual (RR037A, Takara). Real-time polymerase chain reaction (PCR) was performed by utilizing a miRNA-specific forward primer and a universal reverse primer (RiboBio, China), and the thermal cycle profile was: incubation at 95°C for 30 s, by 40 cycles at 95°C for 5 s and at 60°C for 30 s. The gene expression level was detected using the TB Green™ Premix Ex Taq™ II (RR820A, Takara), and the expression of miRNAs was normalized to those of U6.

### Generation of Cell Lines With Stable Overexpression of miR-3613-3p

miRNA-3613-3p pre-miRNA lentiviruses were obtained from GeneChem Biotechnology (Shanghai, China). MDA-MB-231 cells or MDA-MB-468 cells were cultured at 3 × 10^5^ cells/well in 6-well plates. After being incubated for 24 h, the cells were transfected with miRNA-3613-3p pre-miRNA lentiviruses and control sequences following the manufacturer’s instructions. Selection was carried out with puromycin (5 μg/ml, Sigma) in cell culture media for 48 h after transfection. The packaged lentiviruses were named Lv-miR-3613-3p. The empty lentiviral vector Lv-luc was used as a control. Cell lysates were collected, and RT-PCR was performed to detect miRNA expression.

### Cell Viability and Cell Cycle Assays

MTT (Sigma) assay was performed by the following instructions. Cells were seeded and treated with vehicle. MTT was added to the medium to a final concentration of 0.5 mg/ml, and then the medium was removed, and 0.2 ml DMSO was added. After incubation for 30 min at room temperature, the absorbance of the converted dye was measured at 570 nm using a Synergy spectrophotometer. Cell viability was determined by scanning with a micro-plate reader at 570 nm. For cell cycle analysis, cells were washed once in PBS, trypsinized, pelleted at 1,200 ×g, and rinsed once in 2 ml of cold PBS. After centrifugation, cells were slowly resuspended in 2 ml of cold 75% ethanol, after fixation for 12 h at −20°C. Cells were washed in 1 ml PBS after 30-min digestion by RNase (10 mg/ml) at 37°C and stained with 50 μg/ml propidium iodide (PI, Sigma) for 30 min at 4°C. Cells were analyzed for cell cycle stage by flow cytometry.

### EdU Incorporation Assays

MDA-MB-231 cells were transfected with miRNA mimics in 96-well plates. Forty-eight hours after transfection, 5-ethynyl-2′-deoxyuridine (EdU, 100 mM) (Cell Light EdU DNA imaging kit, Guangzhou RiboBio, China) was added, and the cells were cultured for an additional 2 h. The cells were then stained according to the following protocol: The EdU medium mixture was discarded, with 4% paraformaldehyde added for cell fixation at room temperature for 30 min. It was then washed with glycine (2 mg/ml) for 5 min in a shaker, and 0.2% Trion X-100 was added. After 10 min, the sample was washed with PBS two times, and click reaction buffer (Tris–HCl, pH 8.5, 100 mM; CuSO4, 1 mM; Apollo 550 fluorescent azide, 100 mM; ascorbic acid, 100 mM) was added, then care is taken to protect them from exposure to light. After 30–40 min, the samples were washed with 0.5% Triton X-100 three times, stained with Hoechst (5 mg/ml) for 30 min at room temperature, and washed with 0.5% Triton X-100 five times. In the end, 150 ml PBS was added. Images were taken and analyzed by ChemiDocTm XRS Molecular Imager system (Bio-Rad, USA). The rate of EdU-positive cells was by the following equation: Rate of EdU-positive cells = (EdU-labeled cells)/(Hoechst-stained cells) × 100%.

### *In Vitro* Colony Formation Assays

For the colony formation assay, 1,000 cells were placed into each well of a six-well plate and incubated at 37°C for 2 weeks replacing media/drug every 3–4 days. Wells were treated with Palbociclib: 0, 100, 250, 500, 750, 1,000, 1,500, 2,000 nmol/l. Colonies were fixed and stained in a dye solution containing 0.1% crystal violet and 20% methanol, and the number of colonies was counted.

### Western Blotting

Protein extraction and Western blotting were performed according to standard procedures. Primary antibodies were used as follows: anti-c-MYC (1:1,000, Proteintech), anti-EZH2, anti-SMAD2, anti-RB, anti-CDK4, anti-CDK6, anti-CCND1, anti-CCND2 and anti-CCND3 (1:1,000, Cell Signaling Technology) and anti-Actin (1:3,000, Sigma). The membrane was then incubated with appropriate horseradish peroxidase-conjugated secondary antibodies (1:3,000 Cell Signaling Technology). The intensity of protein bands was quantified by using the Image-J software package.

### Immunofluorescence

MDA-MB-231 and MDA-MB-468 cells were transfected with miR-3613-3p mimics or control. For SMAD2, EZH2, and K9MH3 staining, cells were fixed in 4% paraformaldehyde, permeabilized in 0.1% Triton X-100, and probed with an anti-SMAD2, anti-EZH2 antibody (1:200, Cell Signaling Technology) and anti-K9MH3 antibody (1:200, GeneTex). To detect nuclei, cells were co-stained with 40-6-diamidino-2-phenylindole (DAPI; Invitrogen). Cells were observed and pictures were taken by using a confocal microscope (Leica SP5).

### Dual-Luciferase Assay

MCF7 and MDA-MB-231 cells were plated into 24-well plates and could adhere and grow overnight at 37°C with 5% CO_2_. The following day, the cells were co-transfected with pLightSwitch-3UTR GoClone vectors, and 50 nM of miRNA precursors (premiR mimics, Ambion) using Lipofectamine TM 2000 (Invitrogen). MDA-MB-231 cells were transfected with 10 ng of the psiCHECK-2 construct along with 15 pmol of the miR-362-5p mimic or control by using Lipofectamine 3000 (Invitrogen). After 48 h, the cells were lysed, and the firefly and Renilla luciferase activities were measured with the Dual-Luciferase Reporter Assay System (Promega). Each fragment containing the putative miRNA-binding sites was cloned into the psiCHECK-2 vector at the immediate downstream of the Renilla luciferase gene. The results were presented as the ratio of Renilla luciferase activity to firefly luciferase activity.

### Senescence Associated β-Galactosidase (SA-β-gal) staining

Senescence associated *β*-Galactosidase (SA-*β*-Gal) activity in tumor cells was detected according to the kit protocols. Briefly, tumor cell lines were transfected with or without plasmids and cultured for 3 or 5 days. Cells were washed in PBS (pH 7.2), fixed in 3% formaldehyde, and followed by incubating overnight at 37°C with freshly prepared SA-*β*-Gal staining solution. The stained cells were washed with H2O and examined with a microscope. For some experiments, SA-*β*-Gal+ populations were determined in the transfected tumor cells after exposure to various inhibitors or combined transfection with microRNA mimics. The treated tumor cells were then detected for SA-*β*-Gal expression.

### Immunohistochemistry

Paraffin-embedded xenografts or patients’ tissue samples were incubated for 2 h at 56°C for deparaffinization. Antigens were retrieved by microwave treatment in citrate buffer for 15 min to restore antigenicity. After peroxidase activity was blocked with 3% H2O2/methanol for 10 min, sections were incubated with normal goat serum for 20 min to block non-specific antibody binding sites. Sections were incubated with the primary antibodies for 1 h at 25°C and then with biotinylated anti-rabbit/mouse IgG and peroxidase-labeled streptavidin for 10 min each. The following primary antibodies were used: Rabbit anti-EZH2, anti-SMAD2, anti-RB, anti-CDK4 and anti-CCND1 (1:100, Cell Signaling Technology), anti-CCNE1 (1:100, R&D Systems).

### microRNA Target Prediction

The microRNA databases, miRBase (http://microrna.sanger.ac.uk/), microT-CDS (http://diana.imis.athena-innovation.gr/) and TargetScan (http://www.targetscan.org/index.html) were used to identify potential microRNA targets.

### Xenograft Grouping and Tumor Growth Assay

The animals were divided into four groups in terms of treatment protocols. In group one, cells injected had stable overexpression of miR-3613-3p; in group two, cells had overexpression of miR-3613-3p and were then treated with Palbociclib. In group three, control plasmids were transfected. In group four, cells were transfected with control plasmids and treated with Palbociclib. The cell line (MDA-MB-231) with stable miR-3613-3p overexpression and the vector control cells were used to make animal models. For the subcutaneous tumor growth assay, 5 × 10^6^ cells in 0.1 ml serum-free medium with 0.1 ml Basement membrane matrix was subcutaneously injected into 5-week-old female nude mice (six mice per group). When tumors reached a size of approximately 100 mm^3^, the mice were put on a treatment of either water or Palbociclib (100 mg/kg body weight) oral daily. The mice were monitored daily for palpable tumor formation, and tumor growth was measured twice by using external caliper every week, and tumor size was calculated by using the following equation: V = (length × width^2^)/2. Three weeks after Palbociclib treatment, all mice were sacrificed, and the tumors were dissected out for IHC. All procedures were done by strictly following the guidelines of the Institutional Animal Care and Use Committee at Tongji Medical College, Huazhong University of Science and Technology.

### Statistical Analysis

Data were subjected to Student t test by utilizing GraphPad Prism 5 for the comparison between two groups or to an analysis of variance (ANOVA) with Bonferroni correction for multiple comparisons. Probability less than 0.05 was considered statistically significant, and tests were all two-sided. Histograms were used to present the means, and the error bars indicated the SD.

## Results

### MiR-3613-3p Expression Is Down-regulated in TNBC

**Figure 1:**
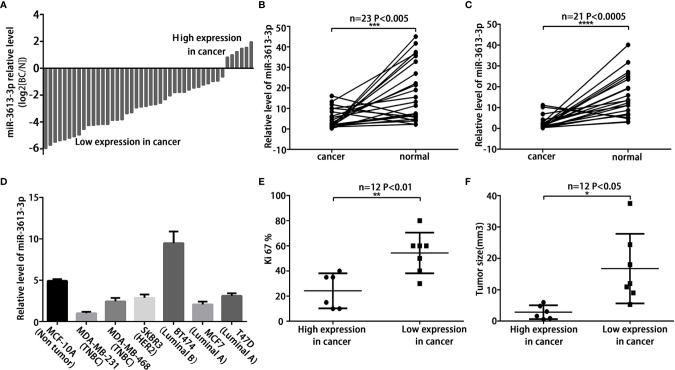
MiR-3613-3p expression is down-regulated in TNBC. MiR-3613-3p expression is down-regulated in TNBC tumors and cells, and correlated with tumor size and Ki-67 LI level. **(A)** Expression of miR-3613-3p in 44 pairs of breast cancer tissues and the corresponding normal tissues. The ratio of miR-3613-3p expression levels in breast cancer tissues to those in normal tissues of each case is indicated by a column. **(B, C)** Expression of miR-3613-3p in HER2 positive breast cancer (n = 23) **(B)** and TNBC (n = 21) **(C)**. **(D)** Relative miR-3613-3p expression levels of seven breast cancer cell lines were examined with the qRT-PCR. Columns, average of three independent experiments. Expression levels of miR-3613-3p were determined by quantitative real-time PCR assay and normalized against an endogenous control (U6 RNA). Data were analyzed using the formula 2−(ΔΔCT). **(E)** Ki67 levels were down-regulated in miR-3613-3p high expression tissues but up-regulated in miR-3613-3p low expression tissues. **(F)** Tumor sizes were smaller in miR-3613-3p high expression tissues. P values were determined by two-tailed t-test, *P < 0.05; **P < 0.01; ***P < 0.001; ****P < 0.0001. Experiments were performed in triplicate.

MiRNA array analysis was performed to compare the miRNA expression profiles in breast cancer tumors and adjacent normal tissues. Significant differences were found in the expression of miR-3613-3p between breast cancer and normal tissues (**Figure S1A**). However, the role and expression level of miR-3613-3p in breast cancer patients remain unknown. To evaluate the findings, we first examined the expression of miR-3613-3p in 44 paired breast cancer tissues by qRT-PCR. Our data showed that miR-3613-3p expression was significantly decreased in breast cancer tissues compared with normal tissues (**Figures 1A**). The finding was further validated in the microarray database OncomiR (OncomiR: http://www.oncomir.org). In bladder urothelial cancer, breast invasive cancer, head and neck squamous cell carcinoma, and uterine corpus endometrial carcinoma, the expression level of miRNA-3613-3p was significantly related to tumorigenesis, and more importantly, only in breast invasive cancer, the expression of miRNA-3613-3p was upregulated in normal tissues (**Figure S1B**). In addition, we found that the expression of miR-3613-3p was more significantly decreased in specimens from HER2-positive breast cancer and triple-negative breast cancer compared to their corresponding adjacent normal tissues (**Figures 1B, C**). Then, the expression of miR-3613-3p was measured in seven breast cancer cell lines. Of note, qRT-PCR revealed that miR-3613-3p was significantly decreased in MDA-MB-231 and MDA-MB-468 (TNBC cell lines) (**Figures 1D**). Detection of breast cancer tumors and cell lines showed that the expression of miR-3613-3p was all significantly reduced in TNBC. Intriguingly, further studies demonstrated that the tumor size was smaller and Ki67 level lower in patients with increased expression of miR-3613-3p in their cancer tissue (**Figures 1E, F****)**. In short, these results suggest that miR-3613-3p might serve as a tumor suppressor gene and that overexpression of miR-3613-3p might be associated with favorable prognosis.

### Overexpression of miR-3613-3p Suppresses the Proliferation, Migration, and Clonogenic Ability in TNBC Cells

**Figure 2:**
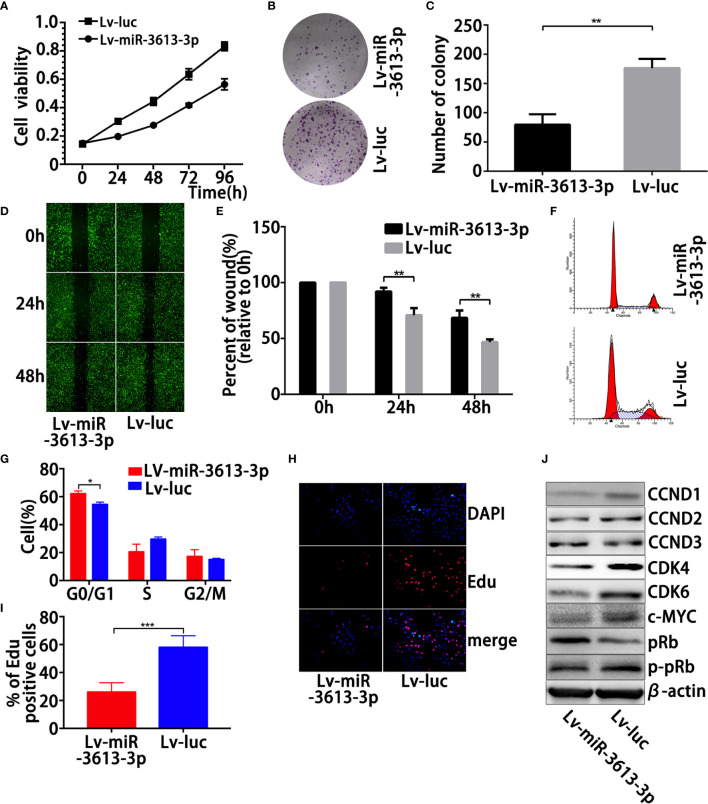
Overexpression of miR-3613-3p suppresses the proliferation, migration, and clonogenic ability induces G0/G1 cell-cycle arrest in TNBC cells. Effects of miR-3613-3p on TNBC cell growth and migration *in vitro*. After generation of miR-3613-3p stable overexpression in MDA-MB-231. **(A)** Cell vitality was evaluated by MTT assay, stably transfected with miR-3613-3p or NC lentivirus. **(B, C)** Colonigenic ability of different MDA-MB-231 cells were tested; generation of miR-3613-3p stable overexpression, the numbers of colony were counted and compared in the diagrams. **(D, E)** Cell migration of different MDA-MB-231 cells was tested using monolayer wound healing assay, generation of miR-3613-3p stable overexpression; the percent of wound closure was counted and compared in the diagrams. **(F, G)** Cell cycle of MDA-MB-231 cells stably transfected with miR-3613-3p or NC lentivirus was analyzed by ﬂow cytometry assay; the percentage of cells in G0/G1, S, and G2/M phase is annotated in each column. **(H, I)** EdU assay of relative DAPI stained cells and EdU add-in cells. MDA-MB-231 cells were stably transfected with miR-3613-3p or NC lentivirus. At least 200 cells were counted per well. **(J)** Western blot analysis of positive cell cycle regulators CCND1, CCND2, CCND3, pRb, p-pRb, c-MYC, CDK4, and CDK6 protein in MDA-MB-231 cells transfected with transfected with miR-3613-3p lentivirus or vector control. P values were determined by two-tailed t-test, **P < 0.01; ****p < 0.0001.

Since parental MDA-MB-231 and MDA-MB-468 cells expressed substantially lower miR-3613-3p, we induced overexpression of miR-3613-3p in these cell lines. Overexpression of miR-3613-3p in MDA-MB-231 and MDA-MB-468 cells transfected with miR-3613-3p mimic and pre-miR-3613 lentiviral expression vector was confirmed by qRT-PCR (**Figures S1C–F**). We employed cell viability assay to determine the generality of the impact of miR-3613-3p regulation on cell growth, and the result showed that the miR-3613-3p overexpression significantly suppressed MDA-MB-231 cell proliferation *in vitro* (**Figure 2A**, **Figure S2A**). Further investigation on the effect of miR-3613-3p on the colonigenic ability of the MDA-MB-231 revealed that miR-3613-3p-transfection had obvious colony-inhibitory effect on the cells compared with control transfectants (**Figures 2B, C**, **Figures S2B, C**). These results indicated that miR-3613-3p might play an inhibitory role in the growth of TNBC tumors. The overexpression of miR-3613-3p in MDA-MB-231 and MDA-MB-468 cells significantly inhibited their migratory abilities (**Figures 2D, E**, **Figures S2D, E**). Our results confirmed that miR-3613-3p could inhibit the migration of TNBC cells.

### Overexpression of miR-3613-3p Induces G0/G1 Cell-Cycle Arrest

To further explore the role of miR-3613-3p in TNBC cell proliferation, we examined the DNA replication and cell cycle in TNBC cells treated with miR-3613-3p mimic. First, flow cytometric detection of the cell cycle exhibited that overexpression of miR-3613-3p significantly augmented the cells in the G0/G1 phase and reduced the cells in the S phase in MDA-MB-231 (**Figures 2F, G**, **Figures S2F, G**). Next, EdU incorporation showed that miR-3613-3p overexpression in these cells led to a reduced EdU incorporation (**Figures 2H, I**, **Figures S2H, I**), suggesting that DNA replication was decreased during the S phase. No effect was observed on apoptosis when MDA-MB-231 was transfected with miR-3613-3p mimic. We further investigated the response of several major cell cycle regulators that were modulated by the miR-3613-3p overexpression. Positive cell cycle regulators mainly work on the G0/G1 phase, and they include, among others, CCND1, CCND2, CCND3, pRb, p-pRB, c-MYC, CDK4, and CDK6. The overexpression of miR-3613-3p reduced the protein levels of cyclins D1, c-MYC, CDK4, and CDK6 (**Figure 2J**, **Figure S2J**). Regulation of these regulators by miR-3613-3p was most likely indirect and reflected its potential in inducing cell-cycle arrest. Therefore, miR-3613-3p might suppress the sustained growth of TNBC cells by regulating cell cycle.

### Overexpression of miR-3613-3p Enhances the Sensitivity of TNBC Cell to Palbociclib by Inducing Senescence

**Figure 3:**
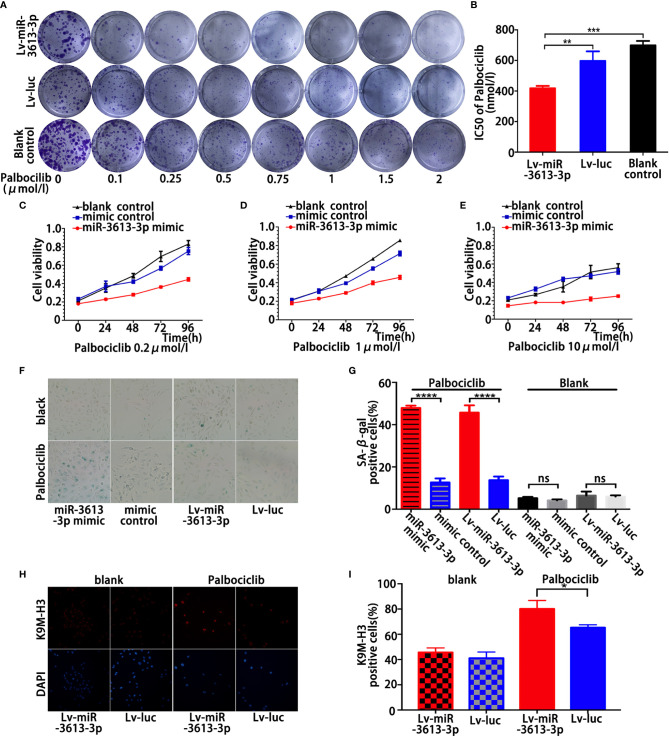
Overexpression of miR-3613-3p enhances the sensitivity of TNBC cell to Palbociclib by inducing senescence. Overexpression of miR-3613-3p enhances the sensitivity of TNBC cell to Palbociclib. **(A, B)** Clonogenic assays of triple MDA-MB-231 cells stably transfected with miR-3613-3p or NC lentivirus grown continuously in increasing concentrations of CDK4/6 inhibitor palbociclib **(A)** and the corresponding IC50 of palbociclib **(B)**. **(C–E)** Cell vitality of MDA-MB-231 cells transfected with miR-3613-3p mimics or mimic control was evaluated by MTT assay treated with the increasing concentrations of CDK4/6 inhibitor palbociclib. **(F, G)** SA-b-galactosidase staining **(F)** and percentage of SA-b-galactosidase positive cells **(G)** among MDA-MB-231, transfected with miR-3613-3p mimics or mimic control and infected with miR-3613-3p lentivirus or vector control, each treated with 5 μM palbociclib. **(H, I)** Immunofluorescence staining of MDA-MB-231 cells transfected with miR-3613-3p mimics or mimic control and infected with miR-3613-3p lentivirus or vector control, using anti-trimethyl K9 histone H3 antibody (K9M-H3, upper panel) or DAPI (lower panel). P values were determined by two-tailed t-test, **p < 0.01; ***p < 0.001; ****p < 0.0001; ns, No statistical significance.

This study showed that miR-3613-3p inhibited the proliferation of TNBC cells by inducing cell-cycle arrest with the expression of CDK4, CDK6, and CCND1 being decreased obviously. Palbociclib, formerly known as PD0332991, is a specific inhibitor of CDK4/6. Palbociclib was demonstrated to work mostly on ER-positive breast cancer cells with luminal features. However, recent studies have found that some TNBCs might be sensitive to CDK4/6 inhibition (22). Therefore, we investigated whether the cells with miR-3613-3p overexpression in TNBC were sensitive to CDK4/6 inhibition. Clonogenic assays were performed on a group of TNBC cell lines with miR-3613-3p overexpression and on a group of TNBC cell lines without miR-3613-3p overexpression, after treatment with Palbociclib. The TNBC cells with miR-3613-3p overexpression were highly sensitive to Palbociclib compared to the TNBC cells without miR-3613-3p overexpression (**Figure 3A**). Meanwhile, IC50 of Palbociclib significantly reduced in miR-3613-3p overexpression cells (**Figure 3B**). Cell viability assay was conducted to examine the effect of miR-3613-3p on the proliferation of MDA-MB-231 cells after Palbociclib treatment. The results suggested that the proliferation rate was significantly decreased by Palbociclib in MDA-MB-231 cells transfected with miR-3613-3p mimic (**Figures 3C** **–E**), and the inhibitory effect was positively correlated with the drug dose. These data indicated that overexpression of miR-3613-3p could, to some degrees, enhanced the sensitivity of TNBC cells to Palbociclib.

Next, inhibition of CDK4/6 with large dose of Palbociclib highly increased the activity of SA-b-Gal, a marker of cellular senescence, in the stably transfected MDA-MB-231 cells (**Figures 3F, G****)**. Other markers of senescence were also detected, such as global chromatin modifications; tri-methylated lysine 9 of histone H3 (K9M-H3) was also greatly increased in MDA-MB-231 cells with stable miR-3613-3p overexpression (**Figures 3H, I****)**. Taken together, these data strongly suggested that overexpression of miR-3613-3p enhances the sensitivity of TNBC cells to Palbociclib by eliciting senescence. Our results were consistent with previous findings that FOXM1 and EZH2 possess senescence-suppressing activity.

### MiR-3613-3p Directly Targets SMAD2 and EZH2

**Figure 4:**
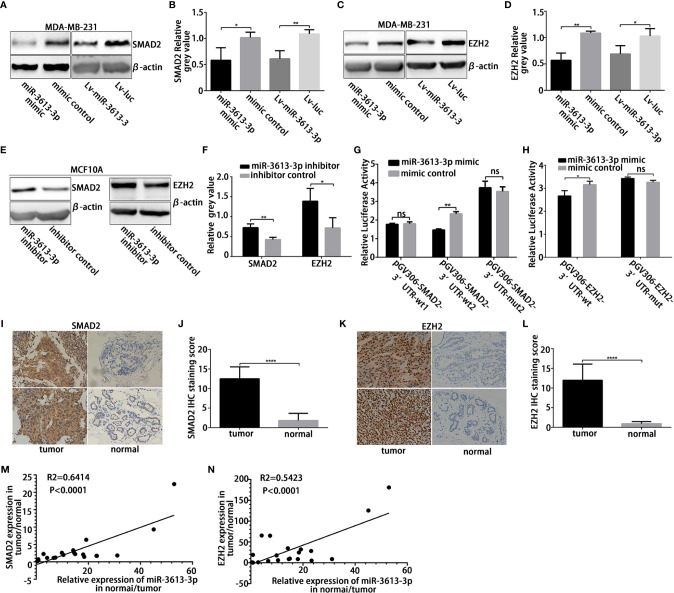
MiR-3613-3p directly targets SMAD2 and EZH2. MiR-3613-3p directly targets the 3′untranslated region (UTR) of SMAD2 and EZH2. **(A, B)** Western blot to analyze SMAD2 protein levels in MDA-MB-231 cell lines when transfected with miR-3613-3p mimic or mimic control and stably transfected with miR-3613-3p or NC lentivirus. **(C, D)** Western blot to analyze EZH2 protein levels in MDA-MB-231 cell lines when transfected with miR-3613-3p mimic or mimic control and stably transfected with miR-3613-3p or NC lentivirus. **(E, F)** Western blot to analyze SMAD2 and EZH2 protein levels in MCF10A cell lines when transfected with miR-3613-3p inhibitor or inhibitor control. **(G, H)** Firefly luciferase activity analysis of SMAD2 3′-UTR and EZH2 3′-UTR performed after co-transfection with SMAD2-wild type **(G)**, EZH2-wild type **(H)** or vector-mutant pGL3 constructs and miR-3613-3p mimic MDA-MB-231 cell lines. **(I–L)** Semi-quantitative analysis and the representative images (×400) of IHC staining for SMAD2 **(I, J)** and EZH2 **(K, L)** protein in 20 paired TNBC FFPE tissues. **(M)** The correlation between miR-3613-3p and SMAD2 protein expression in 20 paired TNBC tissues. **(N)** The correlation between miR-3613-3p and EZH2 protein expression in 20 paired TNBC tissues. P values were determined by two-tailed t-test, *p < 0.05, **P < 0.01; ***P < 0.01, ****p < 0.0001.

To further explore the molecular mechanism by which miR-3613-3p mediates pathological events in TNBC cells, we tried to identify the potential targets of miR-3613-3p by searching bioinformatic databanks, including TargestScan, miRDB, MicroT-CDS, and miRTarBase. SMAD Family Member 2 (SMAD2) and Enhancer of Zeste Homolog 2 (EZH2) are two important genes that play roles in the cell cycle transition and cancer proliferation. They harbor a potential binding site within the 3′UTRs of miR-3613-3p. mRNAs of these two target genes have perfect matches in their 3′UTRs with the seed region (nucleotides 2–8) of miR-3613-3p (**Figures S3A, B**). Since there are many potential binding sites for SMAD2 3′UTRs, we constructed two wild-type fragments of SMAD2 3′UTR potential binding region wt1 and wt2. Predicted miR-3613-3p binding sites in the SMAD2 and EZH2 mRNAs were less conserved among mammals. To validate that these genes are regulated by miR-3613-3p, we first examined the effect of miR-3613-3p on SMAD2 and EZH2 expression. Although miR-3613-3p overexpression did not change SMAD2 and EZH2 mRNA levels (**Figures S3C, D**), the protein levels of SAMD2 and EZH2 were obviously reduced in MDA-MB-231 (**Figures 4A–D**) and MDA-MB-468 cells (**Figures S4A–D**). Furthermore, the expression levels of Cyclin A, Cyclin E, C-MYC, PCNA, and MCM7, target genes of Rb, were detected by qPCR, and the results showed that the expression levels of Cyclin E and C-MYC decreased after overexpression of miRNA-3613-3p, while the expression levels of Cyclin A, PCNA and MCM7 did not change significantly (**Figure S3E**). Meanwhile, silencing miR-3613-3p could increase SMAD2 and EZH2 protein levels in MCF-10A cells (**Figures 4E, F****)**. In addition, immunofluorescence showed that the expression levels of SAMD2 and EZH2 were also decreased in TNBC cells (**Figures S4E, F**). These findings suggested that miR-3613-3p targets SMAD2 and EZH2 through translational inhibition. Then, to further confirm the direct binding and targeting by miR-3613-3p in MDA-MB-231 cells, we constructed Renilla luciferase reporters containing either wild type or mutated 3′UTRs of these two target genes respectively. We found that miR-3613-3p overexpression significantly inhibited luciferase activity in MDA-MB-231 cells with wild-type vectors but not in those with mutant reporter genes (**Figures 4G, H****)**. These results suggested that these sites are critical for miR-3613-3p binding and mediate its regulation.

Then, we immunohistochemically detected the expression of SMAD2 and EZH2 in 20 pairs of TNBC samples and paired paracancer specimens and found that the expression levels of SMAD2 and EZH2 were significantly increased in cancer tissues (**Figures 4I–L**). At the same time, to clarify the correlation between miR-3613-3p and SMAD2/EZH2 in breast cancer, we tested tissue samples from cancer lesions and adjacent non-cancerous areas by using qRT-PCR and Western blotting. We found that miR-3613-3p was negatively correlated with the expression of SMAD2 and EZH2 in the breast cancer samples (**Figures 4M, N****)**. This finding gave further evidence that SMAD2 and EZH2 are regulated by miR-3613-3p in TNBC.

### MiR-3613-3p Inhibits the Growth of TNBC Cells and Enhances Their Sensitivity to Palbociclib *via* SMAD2/EZH2

**Figure 5:**
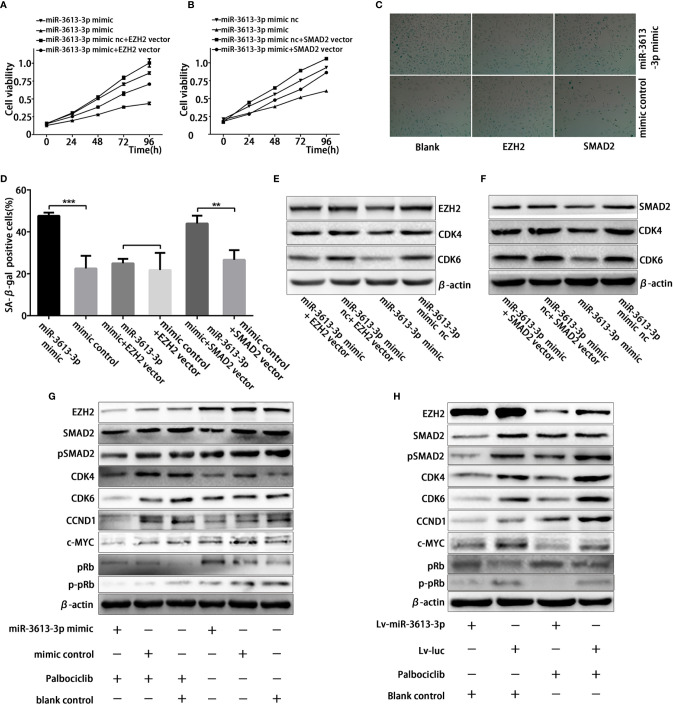
MiR-3613-3p inhibits the growth of TNBC cells and enhances their sensitivity to Palbociclib *via* SMAD2/EZH2. MiR-3613-3p inhibits the growth of TNBC cells and enhances their sensitivity to Palbociclib *via* SMAD2/EZH2. **(A, B)** Cell vitality of MDA-MB-231 cells transfected with miR-3613-3p mimics or mimic control was evaluated by MTT assay, that restoration of EZH2 **(A)** or SMAD2 **(B)** respectively. **(C, D)** SA-b-galactosidase staining **(C)** and Percentage of SA-b-galactosidase positive cells **(D)** among MDA-MB-231 that restoration of EZH2 or SMAD2 respectively transfected with miR-3613-3p mimic or mimic control treated with vehicle or Palbociclib 5 μmol for 48 h. **(E)** Western blotting analysis of EZH2, CDK4, and CDK6 expression in MDA-MB-231 cells that restored EZH2 transfected with miR-3613-3p mimics or mimic control. **(F)** Western blotting analysis of SMAD2, CDK4, and CDK6 expression in MDA-MB-231 cells that restored SMAD2 transfected with miR-3613-3p mimics or mimic control. **(G)** Western blot analysis of EZH2, SMAD2, pSMAD2, CDK4, CDK6, CCND1, c-MYC, pRb, p-pRb and *β*-actin protein in MDA-MB-231 cells transfected with miR-3613-3p mimics or mimic control treated with vehicle or Palbociclib 5 μmol for 48 h. **(H)** Western blot results of EZH2, SMAD2, pSMAD2, CDK4, CDK6, CCND1, c-MYC, pRb, p-pRb and *β*-actin protein in MDA-MB-231 cells infected with miR-3613-3p lentivirus or vector control treated with vehicle or Palbociclib 5 μmol for 48 h. P values were determined by two-tailed t-test, **P < 0.01; ***P < 0.01.

One miRNA tends to regulate multiple mRNA targets under specific cellular context. To investigate whether miR-3613-3p regulates the growth of TNBC cells and enhances their sensitivity to Palbociclib *via* the targets we identified, we first performed cell viability assay to assess the roles of the targets in cell proliferation. Our results demonstrated overexpression of miR-3613-3p resulted in a markedly decreased cell viability, which could be partially rescued by the overexpression of SMAD2/EZH2 in MDA-MB-231 cell lines (**Figures 5A, B****)**. Detection of SA-b-Gal activity revealed that the overexpression of miR-3613-3p led to an increased activity of SA-b-Gal in MDA-MB-231 cells after treatment with Palbociclib. Moreover, EZH2 overexpression could partially rescue the increase in MDA-MB-231 cells (**Figures 5C, D****)**. In addition, Western blotting showed that the overexpression of SMAD2/EZH2 counteracted the miR-3613-3p-induced inhibitory effect of SMAD2/EZH2 expression at protein levels and, meanwhile, upregulated cell cycle-related proteins (**Figures 5E, F****)**. More importantly, we found that the expression levels of EZH2, CDK4, CDK6, CCND1, p-pRb, and c-MYC protein were significantly decreased in Palbociclib-treated MDA-MB-231 cells with miR-3613-3p overexpression (**Figures 5G, H****)**.

### Overexpression of miR-3613-3p Suppresses Tumorigenicity and Enhances the Sensitivity of TNBC Cells to Palbociclib In Vivo

**Figure 6:**
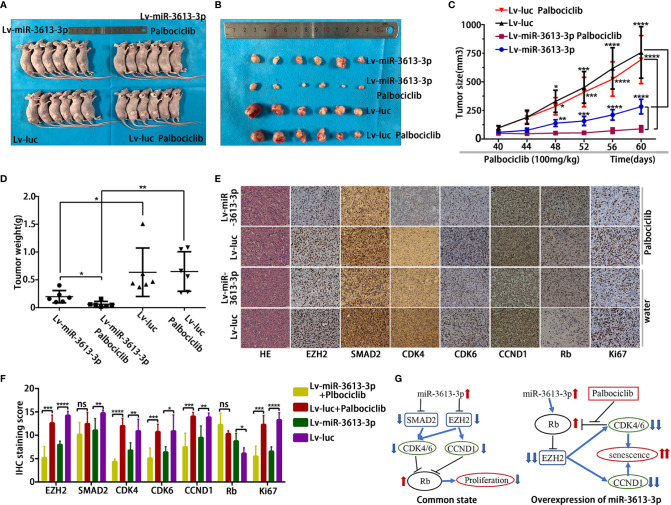
Overexpression of miR-3613-3p suppresses tumorigenicity and enhances the sensitivity of TNBC cells to Palbociclib *in vivo*. Effect of miR-3613-3p overexpression on the Palbociclib sensitivity of TNBC cells *in vivo*. **(A, B)** Xenografts of MDA-MB-231 cells infected with miR-3613-3p lentivirus or vector control, treated daily with vehicle (n = 6) or Palbociclib (n = 6) for 3 weeks. **(C, D)** Tumor growth curves and the mean tumor weight of MDA-MB-231 cells infected with miR-3613-3p lentivirus or vector control treated with vehicle or Palbociclib *in vivo*. **(E)** Representative images of EZH2, SMAD2, CDK4, CDK6, CCND1, Rb, and Ki67 IHC staining and HE staining in paraffin sections from the four paired MDA-MB-231 tumor xenografts (×400). **(F)** Semi-quantitative analyses of IHC results for EZH2, SMAD2, CDK4, CDK6, CCND1, Rb, and Ki67 expression from the four paired MDA-MB-231 tumor xenografts. **(G)** Diagram of the mechanism by which miRNA-3613-3p exerts its inhibitory effect. Error bars in **(C)** indicate standard error of mean (SEM). P values were determined by two-tailed t-test, ns, not significant; *P < 0.05; **p < 0.01; ***p < 0.001; ****p < 0.0001.

To understand the potential *in vivo* effects of miR-3613-3p on the growth of breast cancer cells, an equal number (5 × 10^6^) of MDA-MB-231 cells transfected with pre-miR-3613 lentiviral expression vectors or the pre-control vectors were subcutaneously injected into female BALB/c nude mice. After transfection and drug treatment (group two), the overexpression of miR-3613-3p significantly inhibited tumor growth as compared with those transfected with pre-control vectors (group four) and those without drug treatment (group one) (**Figures 6A, B****)**. Moreover, tumor sizes and weight were greatly reduced in miR-3613-3p overexpression mice treated with Palbociclib (group two) (**Figures 6C, D****)**. IHC showed that the expression levels of EZH2, CCNE1, CDK4, CDK6, and Ki67 were down-regulated in group two (**Figures 6E, F****)**. We found the expressions of these markers were also decreased but the extents were less than those in group one. These results provided further evidence that miR-3613-3p overexpression could inhibit tumor growth and enhance the sensitivity of TNBC cells to Palbociclib by targeting EZH2, CDK4, CDK6, and CCND1.

Mechanistically, under common conditions, overexpression of miR-3613-3p can regulate the expression of EZH2 and SMAD2 by targeting, thereby reducing the expression of cyclin CDK4/6 and CCND1, resulting in increased Rb protein and inhibiting cell proliferation. In the state of overexpressing miRNA-3613-3p, the Rb protein increased, and after treatment with Palbociclib, the phosphorylation of Rb by CDK4/6 was inhibited, which can lead to a further reduction in the expression of EZH2 and further reduce the expression of CDK4/6 and CCND1, triggering cell senescence (**Figure 6G**).

## Discussion

Poor clinical outcome of TNBC has been ascribed to lack of specific targets (23). Recently, several studies have focused on identifying the targets that can help predict prognosis and enhance the sensitivity of malignant cells to anti-cancer agents (24). With many cancers, microRNAs serve as biomarkers that are easily detectable and facilitate the prediction of prognosis and judgement of response to therapy. Mounting evidence suggests that miR-3613-3p is a functionally important miRNA and is potentially implicated in a wide array of important biological processes and critical pathways associated with cardiovascular pathology (25). MiR-3613-3p was found to be repressed in neuroblastoma cells, possibly *via* the regulation of MIPIP1 by cleaving its precursors (26). Plasma miR-3613-3p is a specific biomarker of lung adenocarcinoma (27). Nonetheless, each type of tumor has a distinct microRNA signature that distinguishes it from normal tissues and other cancer types. Moreover, the underlying mechanism responsible for miR-3613-3p dysregulation in invasive breast cancer remains unknown. In this study, we showed, for the first time, that miR-3613-3p was significantly decreased in tumor tissues of TNBC patients and TNBC cell lines. What is more, overexpression of miR-3613-3p substantially inhibited the migration and proliferation of TNBC cells *in vitro* and tumor proliferation *in vivo* by targeting SMAD2 and EZH2. The miR-3613-3p could suppress SMAD2 and EZH2 expression by directly binding to their 3′UTR. Moreover, we examined the correlation between the expression level of miR-3613-3p and the clinical features of paired breast cancer tissues. Our study revealed a novel mechanism by which miR-3613-3p regulates SMAD2/EZH2 network in TNBC and demonstrated that miR-3613-3p functions as a tumor-suppressive miRNA in TNBC.

Identification of target genes of miR-3613-3p is critical for the understanding of its role in tumorigenesis and is important for identifying novel therapeutic targets. To date, two targets of miR-3613-3p, DFFB and APAF1, have been identified (26). In this study, we found out that SMAD2 and EZH2 were target genes of miR-3613-3p. SMAD2 is a member of the SMAD family and a key element in the TGF-b signaling pathway. In normal cells and at early stages of breast carcinoma, TGF-*β* acts as a tumor suppressor by inducing cell-cycle arrest and apoptosis. Nevertheless, more aggressive breast carcinoma cells often become resistant to the inhibitory effect of TGF-*β* growth signaling pathway. As a consequence, TGF-*β* switches to a promoter of tumor progression and metastasis (28).

In this study, we found that the expression of SMAD2 at the protein level was upregulated in TNBC, and SMAD2 exhibited oncogenic phenotypes. Moreover, we demonstrated that SMAD2 was repressed at translational levels by miR-3613-3p, suggesting it is a direct target of miR-3613-3p. Previous studies also showed SMAD2 had therapeutic effects, and that blocking SMAD2 could suppress TGF-*β*-induced tumorigenesis and epithelial–mesenchymal transition (EMT). Several recent studies reported that SMAD2 promoted the tumor progression by upregulating the CDK2, CDK4, and cyclin E in metastatic breast cancer cells (29). On the other hand, EZH2 protein levels were strongly associated with breast cancer aggressiveness (30). EZH2 is controlled by E2F transcription factors, which are at the downstream of the retinoblastoma protein (Rb) pathway (31). The genetic deletion of EZH2 resulted in down-regulation of cyclin D1 in breast cancer (32) and in up-regulation of p21 in ovarian cancer (33), thereby causing cell-cycle arrest at the G1/S phase. Therefore, overexpression of miR-3613-3p inhibited cell proliferation by regulating cell cycle.

Dysregulation of the cell cycle is involved in tumorigenesis (34). Many miRNAs are implicated in the regulation of the cell cycle, such as miRNA-181 (35) and miR-4779 (36). In this study, overexpression of miR-3613-3p induced G0/G1 cell-cycle arrest in MDA-MB-231 cells. Previous study showed that overexpression of SMAD2 promoted cervical cancer cell growth by facilitating the G1/S phase transition. CDC25A, an activator of cell cycle regulators, can accelerate the entry of cells into S-phase and mitosis by regulating cyclin E, CDK2, and CDK4 in a Smad2-dependent fashion (29). The genetic deletion of EZH2 reportedly resulted in the down-regulation of cyclin D1 in breast cancer and caused the up-regulation of p21 in ovarian cancer, triggering cell-cycle arrest at the G1/S phase (32). Therefore, we are led to hypothesize that miR-3613-3p can decrease the expression of CDK4, CCND1 and CCNE1 by targeting SMAD2 and EZH2, leading to the arrest of cell cycle.

Previous studies exhibited that, in glioblastoma, there was an EZH2-CDK4/6-pRb-E2F1 signal loop that mediated cell cycle and cellular proliferation (37). We found that the overexpression of miR-3613-3p significantly decreased the protein expression of CDK4, CDK6, CCND1, CCNE1, EZH2, and increased the protein expression of Rb. Therefore, we theorize that increasing the level of miRNA-3613-3p can enhance the sensitivity of TNBC cells to Palbociclib, because the overexpression of miRNA-3613-3p could substantially block the EZH2-CDK4/6-pRb-E2F1 signaling loop and increase the protein expression of Rb, resulting in more phosphorylation inhibition. Our results confirmed that overexpression of miRNA-3613-3p significantly reduced the expression of CDK4, CDK6, c-MYC, and EZH2 and induced cell senescence in TNBC cells treated with Palbociclib, which is consistent with reports that CDK4/6 inhibitors can induce G1 cell-cycle arrest and a phenotype resembling cellular senescence (38). Although, Palbociclib (PD 0332991) has been approved by the US Food and Drug Administration (FDA) on February 3, 2015 as a first-line medicine for advanced post-menopausal ER+, HER2-negative breast cancer, used in combination with letrozole (9). However, it is generally agreed that the early use of a CDK 4/6 inhibitor in younger patients and in those with aggressive, PR-negative and/or symptomatic condition (10). Meanwhile, previous studies showed that the TNBC cells, such as HCC38, MDA-MB-231, SUM-159 cell lines that highly express Rb and LMWE were significantly more sensitive to Palbociclib than the cells with low expression of Rb or LMWE because they can mediate growth inhibition, G1 arrest and senescence in a dose-dependent manner (39). Recent research showed that cell-cycle dynamics determined the response of TNBC cells to CDK4/6 inhibitors. Luminal androgen receptor (LAR) subtype of TNBC was highly sensitive to CDK4/6 inhibition both *in vitro* and *in vivo* (11). Moreover, the CDK4/6 inhibitors, when used in combination with PI3 kinase inhibitor, could serve as a novel therapeutic strategy for specific subtypes of TNBC (40). Therefore, there is a strong rationale to test CDK4/6 inhibitors in TNBC. To the best of our knowledge, this investigation is the first study to reveal that the overexpression of miR-3613-3p can enhance the sensitivity of TNBC cells to Palbociclib. This suggests that clinically some patients with higher expression of miR-3613-3p in their cancer tissues may respond well to Palbociclib.

Our study preliminarily showed the overexpression of miR-3613-3p can enhance the sensitivity of TNBC cells to Palbociclib; some questions have yet to be answered. First, further studies are needed to clarify whether miR-3613-3p mimic can be used for the treatment of TNBC and whether this treatment has adverse effects. Second, more work has to be done to know whether the phenomena we observed in TNBC cells also occur in other subtypes of triple-negative breast cancer cells and non-negative breast cancer cells. Third, further study is warranted to understand the exact mechanisms by which EZH2 works with CDK4/6 in the regulation of TNBC cells.

In summary, our findings suggest that miR-3613-3p acts as a cancer-suppressor miRNA and serves its anti-cancer function by inhibiting SMAD2 and EZH2 in breast cancer. Moreover, our study showed that overexpressing of miR-3613-3p might be a novel strategy for inhibiting the proliferation of breast cancer and might be used as a predictive biomarker for the response of TNBC to Palbociclib.

## Data Availability Statement

The raw data supporting the conclusions of this article will be made available by the authors, without undue reservation.

## Ethics Statement

The studies involving human participants were reviewed and approved by the Institutional Human Ethics Committee of Union Hospital, Tongji Medical College, Huazhong University of Science and Technology. The patients/participants provided their written informed consent to participate in this study. The animal study was reviewed and approved by the Institutional Animal Care and Use Committee at Tongji Medical College, Huazhong University of Science and Technology. Written informed consent was obtained from the owners for the participation of their animals in this study.

## Author Contributions

BZ and JC designed the experiments. YY and HL performed and conceived the experiments, and wrote the paper. RX, YZ, and RZ provided clinical information. JC assisted in improving the quality of language and revising the statistical method. All authors contributed to the article and approved the submitted version.

## Funding

This work was supported by the National Natural Science Foundation of China (nos. 81472487 and 81773197).

## Conflict of Interest

The authors declare that the research was conducted in the absence of any commercial or financial relationships that could be construed as a potential conflict of interest.
