# Supplementary material for: Overexpression of miRNA-3613-3p Enhances the Sensitivity of Triple Negative Breast Cancer to CDK4/6 Inhibitor Palbociclib
Source: Front Oncol. 2020 Nov 27;10:590813. doi: 10.3389/fonc.2020.590813 (PMC7729088; doi:10.3389/fonc.2020.590813)
Supplement: Supplementary file 1 [file DataSheet_1.docx]

**Overexpression of miRNA-3613-3p** **enhances the sensitivity**

**of** **triple negative breast cancer to CDK4/6** **inhibitor Palbociclib**

1. Supplementary Figures 1.
2. Supplementary Figures 2.
3. Supplementary Figures 3.
4. Supplementary Figures 4.


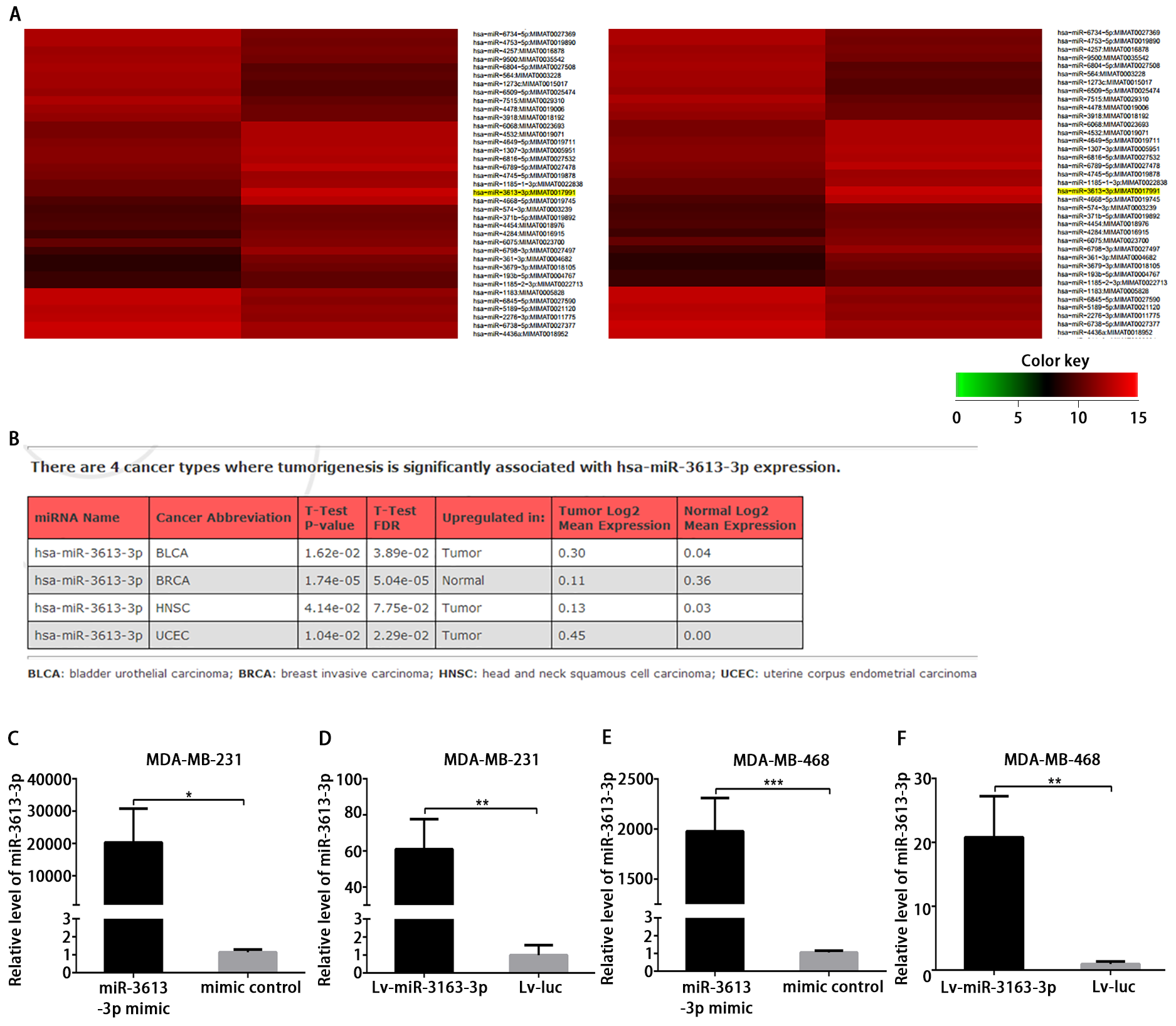


**Supplementary Figures 1.** (A) Microarray analysis demonstrated miRNA-3613-3p was significant different expression in breast cancer samples compared to paired normal tissues. (B) The expression of miR-3613-3p in the microarray database OncomiR of different tumors. (C-F) The relative levels of miR-3613-3p expression. Transfection with miR-3613-3p mimic (C) and pre-miR-3613 lentiviral (D) expression vector were confirmed by qRT-PCR in MDA-MB-231 cells. Transfection with miR-3613-3p mimic (E) and pre-miR-3613 lentiviral (F) expression vector were confirmed by qRT-PCR in MDA-MB-468 cells.


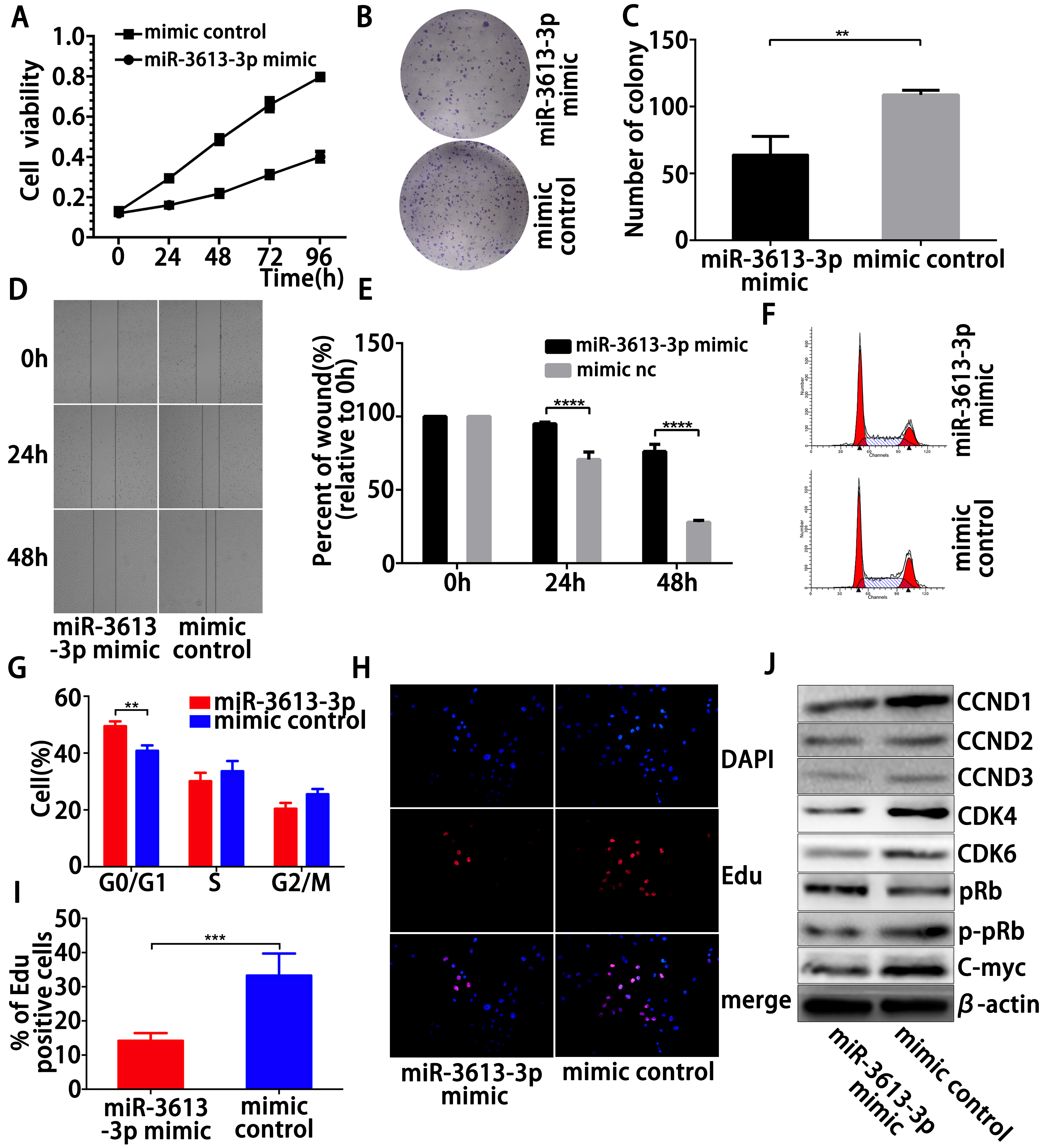


**Supplementary Figures 2.** After transfection with miR-3613-3p mimics in MDA-MB-231. (A) Cell vitality was evaluated by MTT assay, MDA-MB-231 cells transfected with miR-3613-3p mimics. (B-C) Colonigenic ability of different MDA-MB-231 cells were tested, transfection with miR-3613-3p mimics. (D-E) Cell migration of different MDA-MB-231 cells were tested using monolayer wound healing assay, transfection with miR-3613-3p mimics. (F-G) Cell cycle of MDA-MB-231 cells transfection with miR-3613-3p mimics was analyzed by ﬂow cytometry assay, the percentage of cells in G0/G1, S and G2/M phase are annotated in each column. (H-I) EdU assay of relative DAPI stained cells and EdU add-in cells. MDA-MB-231 cells were transfected with miR3613-3p mimics or mimic control. At least 200 cells were counted per well. (J) Western blot analysis of positive cell cycle regulators CCND1, CCND2, CCND3, pRb, p-pRb, c-MYC, CDK4 and CDK6 protein in MDA-MB-231 cells transfected with transfected with miR-3613-3p mimics or mimic control. P values were determined by two-tailed t-test, **, P<0.01; ***, P<0.01.


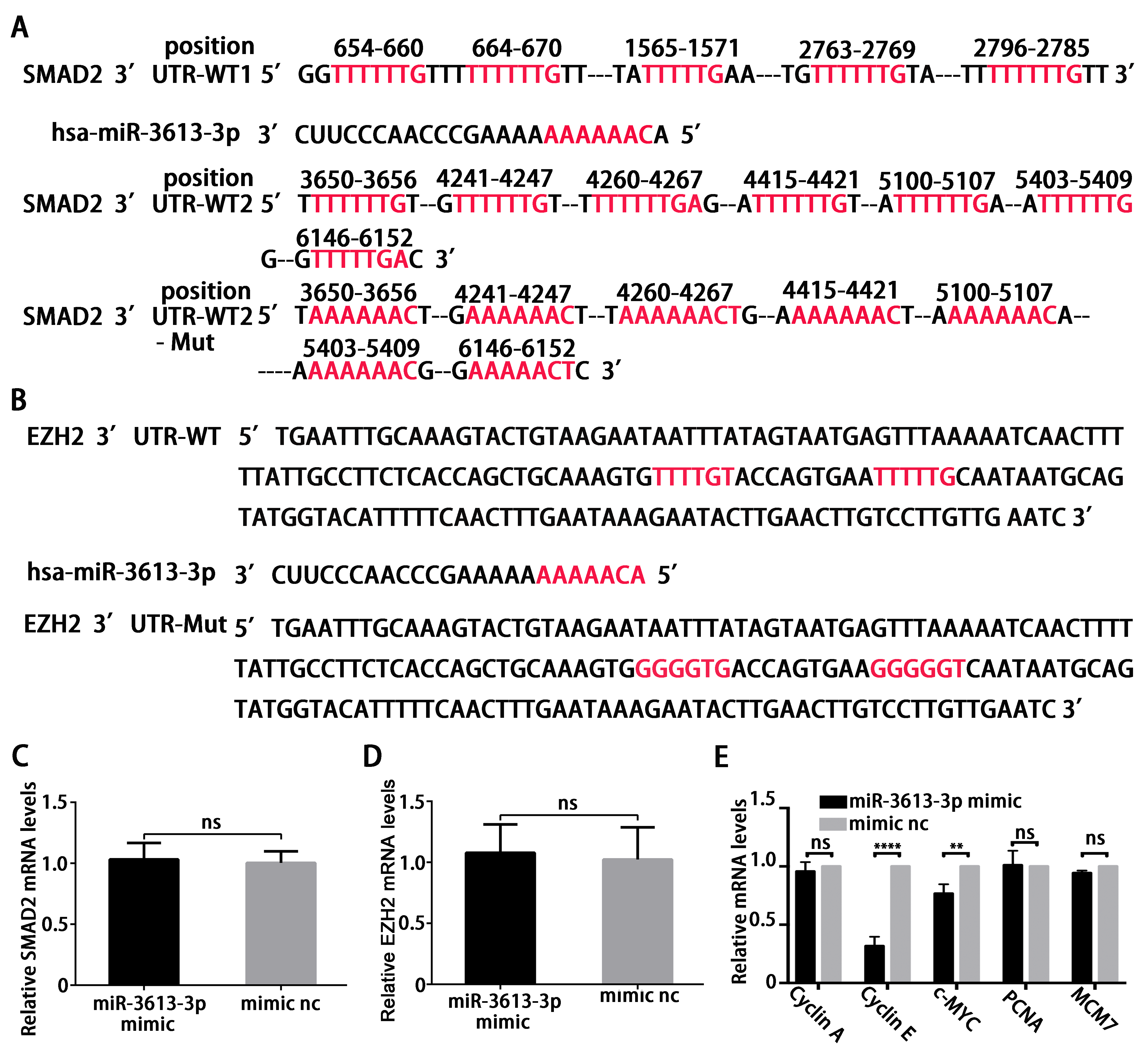


**Supplementary Figures 3.** (A-B) The predicted miR-3613-3p binding site in the 3’UTR of the human SMAD2 (A) and EZH2 (B) genes. Nucleotides 2–7, representing the seed sequence, highlighted in red. (C-D) Real-time PCR for SMAD2 (C) and EZH2 (D) expression after 48 h transfection with control and miR-3613-3p mimics in MDA-MB-231 cells. (E) Real-time PCR for the expression of Cyclin A, Cyclin E, C-MYC, PCNA and MCM7 after 48 h transfection with control and miR-3613-3p mimics in MDA-MB-231 cells.


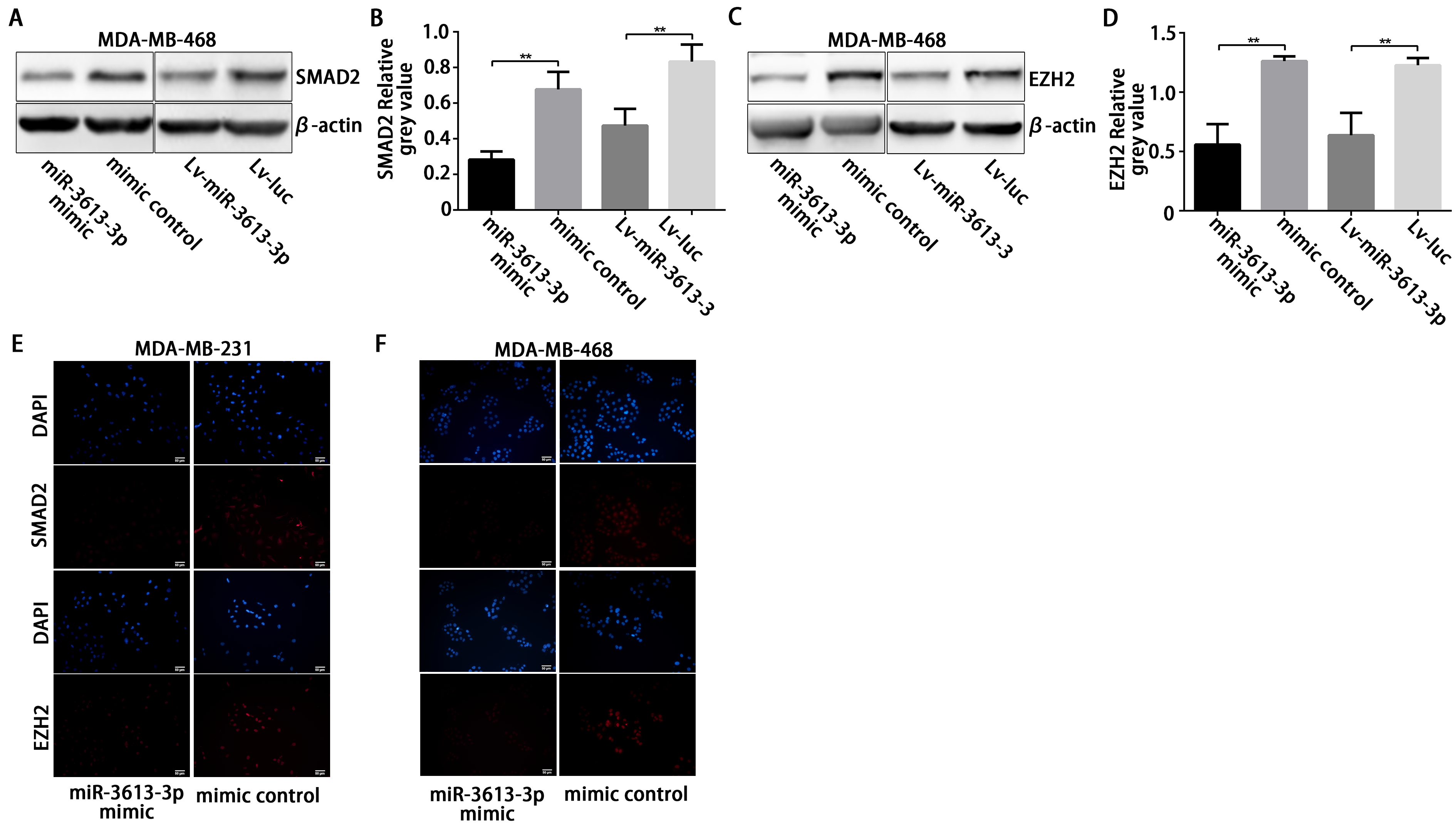


**Supplementary Figures 4.** (A-B) Western blot to analyze SMAD2 protein levels in MDA-MB-468 cell lines when transfected with miR-3613-3p mimic or mimic control and stably transfected with miR-3613-3p or NC lentivirus. (C-D) Western blot to analyze EZH2 protein levels in MDA-MB-468 cell lines when transfected with miR-3613-3p mimic or mimic control and stably transfected with miR-3613-3p or NC lentivirus. (E-F) Expressions of SMAD2 and EZH2 were detected by immunofluorescence in TNBC cell lines that transfected with miR-3613-3p mimic or mimic control.
